# Supplementary material for: Non-Structural Carbohydrates Accumulation in Seedlings Improved Flowering Quality of Tree Peony under Forcing Culture Conditions, with Roots Playing a Crucial Role
Source: Plants (Basel). 2024 Oct 10;13(20):2837. doi: 10.3390/plants13202837 (PMC11511261; doi:10.3390/plants13202837)
Supplement: Supplementary file 1 [file plants-13-02837-s001.zip › plants-3193077-supplementary.pdf]

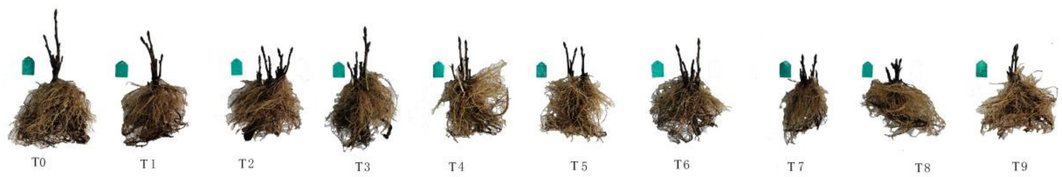

Figure S1. Seedlings morphology of potted tree peony at defoliation stage

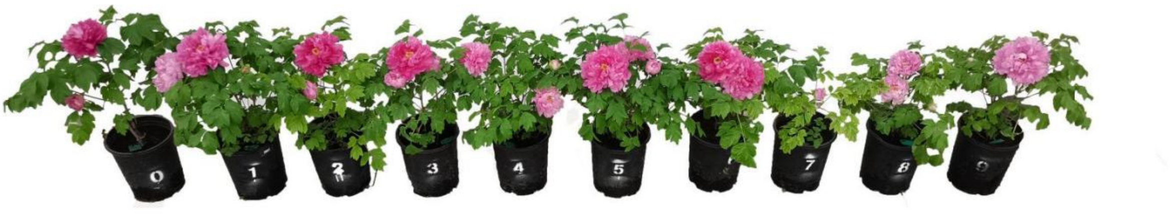

Figure S2. The flowering status of off-season potted tree peony by forcing culture
